# Nutrition management by a multidisciplinary team for prevention of nutritional deficits and morbidity following esophagectomy

**DOI:** 10.1590/1414-431X2023e12421

**Published:** 2023-04-14

**Authors:** Juan Chen, Ai-Lin Luo, Lin Yang, Wei Wang, Xian Zhou, Mei Yang

**Affiliations:** 1 Sichuan University Department of Thoracic Surgery, West China Hospital Chengdu China Department of Thoracic Surgery, West China Hospital, Sichuan University, Chengdu, China

**Keywords:** Multidisciplinary team, Perioperative period, Esophagus cancer, Nutriture, Complication

## Abstract

This study evaluated the effects of perioperative nutrition management by a multidisciplinary team on nutrition and postoperative complications of patients with esophageal cancer. A total of 239 patients with esophageal cancer who underwent esophagectomy and gastric conduit reconstruction for esophageal or esophagogastric junction cancer between February 2019 and February 2020 were included in the study. They were divided into the experimental group (120 patients) and the control group (119 patients) using the random number table method. Control group patients received routine diet management and experimental group patients received perioperative nutrition management by a multidisciplinary team. The differences of nutriture and postoperative complications between the two groups were compared. At 3 and 7 days after surgery, the experimental group patients had higher total protein and albumin levels (P<0.05), shorter postoperative anal exhaust time (P<0.05), lower incidence of postoperative gastrointestinal adverse reactions, pneumonia, anastomotic fistula, hypoproteinemia (P<0.05), and lower hospitalization costs (P<0.05) than the control group. Nutrition management by a multidisciplinary team effectively improved the nutriture of patients, promoted the rapid recovery of postoperative gastrointestinal function, reduced postoperative complications, and reduced hospitalization costs.

## Introduction

Esophageal cancer is a common malignant tumor of the digestive tract, with an incidence ranked 7th and mortality ranked 6th among the most common malignancies worldwide (1). The newly diagnosed esophageal cancer cases in China account for about half of the worldwide cases. In China, the incidence of esophageal cancer ranks 6th and its mortality rate ranks 4th among all malignant tumors (2). Esophagectomy is the cornerstone of curative treatment for esophageal cancer. Previous studies have shown that the perioperative nutritional status of patients with esophageal cancer is related to the occurrence of postoperative complications, and that nutritional status is an independent prognostic factor for esophageal cancer patients (3-5). Patients with esophageal cancer are at high risk for malnutrition due to progressive dysphagia and insufficient food intake (6,7). The nutritional status of patients deteriorates with insufficient postoperative nutritional support, which worsens the prognosis (8). Therefore, the perioperative nutritional management of patients with esophageal cancer is of great importance.

The multidisciplinary team approach has been implemented for various cancers in recent years. Previous studies have shown that nurses play an indispensable role in all aspects of esophageal cancer management (9,10). They are healthcare professionals who share disease-related information, deliver health education, and facilitate patient engagement, and therefore provide the skills individuals need to adhere to the guidelines, thereby enhancing the effects on their health (11). However, studies reporting a multidisciplinary, nurse-led, patient-centered approach to preventing nutritional deficits and morbidity following esophagectomy are sparse and limited. Hence, we performed a randomized controlled trial to investigate the effectiveness of a nurse-led multidisciplinary team approach to nutritional management of patients with esophageal cancer.

## Material and Methods

### Study design

This study was a parallel-group, single-blind, randomized clinical trial conducted at the Department of Thoracic Surgery, West China Hospital, Sichuan University. The study was approved by the Human Participants Committee of West China Hospital of Sichuan University (Ethical number: 2021-355).

### Study population

**Figure 1:**
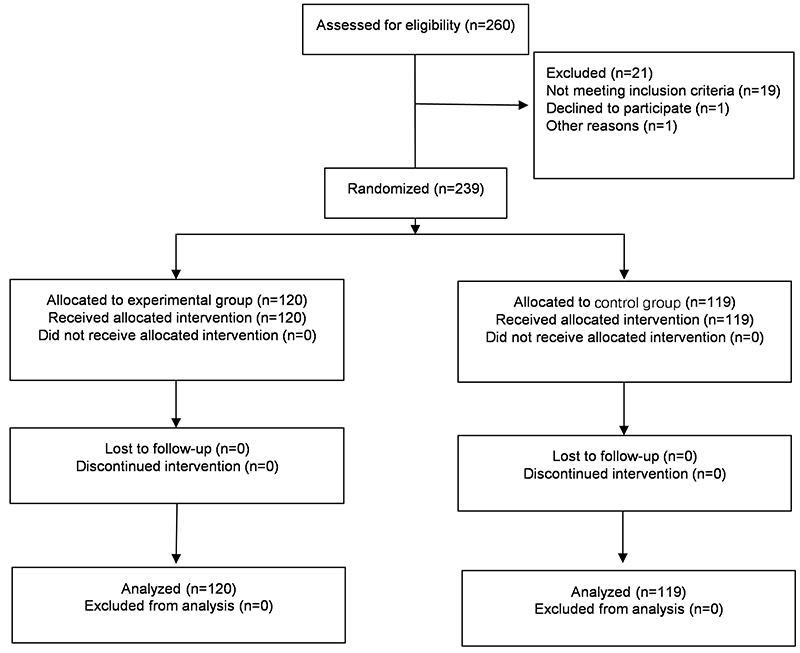
Clinical trial flowchart.

Patients who were diagnosed with esophageal cancer and had chosen to receive esophagectomy at the Department of Thoracic Surgery, West China Hospital, Sichuan University from February 2019 to February 2020 were assessed for trial eligibility. Inclusion criteria were the following: age ≥18 years, preoperative pathological diagnosis of esophageal malignant tumor, the surgical method was thoracic laparoscopy combined with esophageal cancer resection, and voluntary participation in the study by signing an informed consent. Exclusion criteria were other malignant tumors, severe heart, liver, and kidney dysfunction, other diseases of the gastrointestinal tract, or diseases that affect digestion and absorption. The total of 239 patients who met the eligibility criteria were divided into the experimental group (120 patients) and the control group (119 patients) according to the random number table method (Figure 1). There were 97 males and 23 females in the experimental group, with an average age of 63.78±9.13 years and 94 males and 25 females in the control group, with an average age of 64.12±7.91 years.

### Randomization

This study used block randomization with a block size of 6, and the patients were randomly divided into the control group and experimental group by a 1:1 ratio. The first researcher generated a random number table and the second researcher put the random number and group number in opaque envelopes of the same size and color. Each envelope cover had a screening number, which was the serial number of the research object entering the screening. The random assignment was implemented by the third researcher. After the patients were admitted to the hospital, the researcher gave the envelope with the patient's serial number and hospitalization number to the nutrition management team. Randomization, concealment, and allocation were implemented by 3 different researchers, and none of these 3 researchers participated in the follow-up part of the intervention plan. The study subjects and their families were blinded, and the nutrition plan was implemented by the nutrition management team. Follow-up personnel and data statisticians did not know the patients' group.

### Intervention

Patients in the experimental group received perioperative nutrition management by a multidisciplinary team. The multidisciplinary team (MDT) was composed of physicians (1 thoracic surgery chief physician, 1 attending physician), nurses (1 head nurse, 2 nurses in charge, 2 nurse practitioners), 1 nutritionist, and 1 medical record information technician. Before implementation of the intervention, all members of the MDT participated in and determined the perioperative nutrition management plan. The evaluation team (2 nurses in charge and 1 nutritionist) was responsible for the evaluation of the patient's nutritional status after admission, after surgery, and after discharge. The intervention team (1 attending physician, 2 nurses in charge, and 1 nutritionist) was responsible for implementing specific interventions. The data team (2 nurse practitioners and 1 medical record department information technician) was responsible for collecting experimental data and data analysis of the two groups of patients. The head nurse was responsible for collecting feedback and quality control.

**Figure 2:**
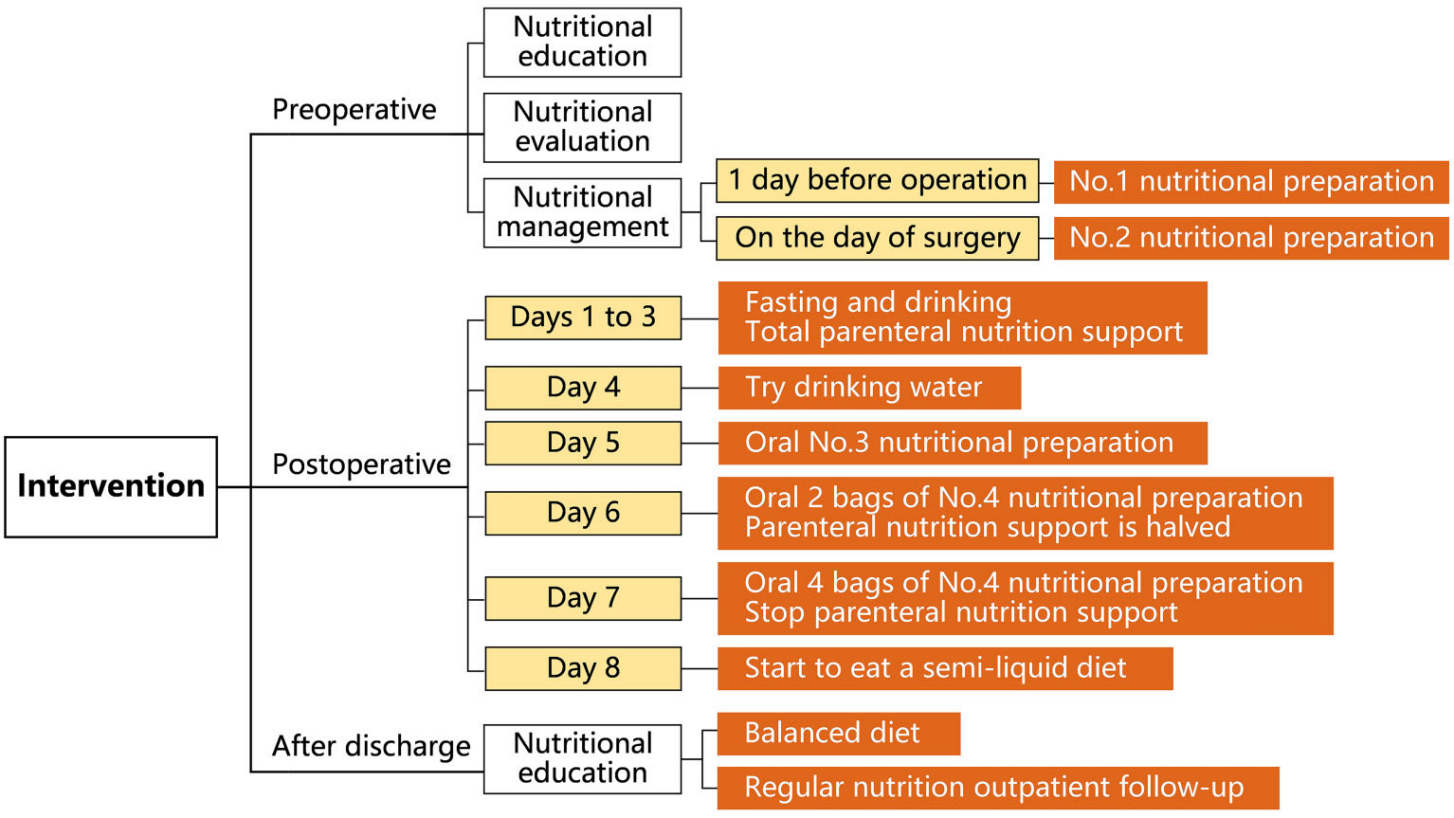
Intervention flowchart.

The MDT perioperative nutrition management included three stages: preoperative, postoperative, and after discharge. The process did not include placement of a gastric tube and enteral nutrition tube. One week before the operation, the evaluation team used the NRS2002 Nutrition Risk Screening Sheet to score the nutritional status of the patients within 24 h after admission. Patients with a total score of <3 were instructed to eat a high-energy, high-protein, and high-vitamin diet. For patients with a total score of ≥3, the Patient-Generated Subjective Global Assessment (PG-SGA) recommended by the Chinese Anti-Cancer Association was used to evaluate nutritional status (12). The intervention team implemented individualized nutrition management of patients based on the evaluation results. One day before the operation, the patients took one sachet of No. 1 nutritional supplement (415 kcal energy, 17 g of protein, 46 g of lipid, 48 g of carbohydrate, 617 mg of potassium, 414 mg of sodium, 387 mg of calcium, and 5 g dietary of fiber in a 300 mL volume) in the morning, at noon, and in the evening. On the day of surgery, the patients took one sachet of No. 2 nutritional supplement (193 kcal energy, 0 g of protein, 0 g of lipid, 48 g of carbohydrate, 0 mg of potassium, 3 mg of sodium, 0 mg of calcium, and 0 g dietary of fiber in a 250 mL volume). At the same time, the intervention team educated patients on nutrition knowledge through various forms of nutrition lectures and educational videos. The information provided included nutritional risks for patients with esophageal cancer, the importance of nutritional support, the usage and precautions of nutritional preparations, etc. (Figure 2).

The patients were not allowed to drink or eat on postoperative days 1 to 3. The patients in the intervention group received total parenteral nutrition support through intravenous infusion of nutrient solutions containing glucose, amino acids, fat emulsions, and electrolytes. On postoperative day 4, the patients tried to drink water and observed whether there were any adverse reactions such as choking, vomiting, chest pain, etc. after drinking the water. If there were no adverse reactions after drinking water, the patients could start orally ingesting No. 3 nutritional supplement (315 kcal of energy, 19 g of protein, 7 g of lipid, 43 g of carbohydrate, 352 mg of potassium, 216 mg of sodium, 206 mg of calcium, and 1 g of dietary fiber) on the 5th day after surgery. The regimen was 15-30 g every 1 to 2 h, mixed with warm boiled water in a ratio of 1:2, taken slowly according to the principle of “from less to more”, while continuing on parenteral nutrition support. According to the food tolerance on the 5th day, on postoperative day 6 patients were given orally 2 sachets of No. 4 nutritional supplement (300 kcal energy, 12 g of protein, 3 g of lipid, 56 g of carbohydrate, 282 mg of potassium, 165 mg of sodium, 90 mg of calcium, and 0 g dietary of fiber). The regimen was 30-45 g every 1 to 2 h, mixed with warm water in a ratio of 1:2, taken slowly according to the principle of starting “from less to more”. At the same time, the amount of parenteral nutrition support was halved. According to the patients' food tolerance on the 6th day, patients were given orally 4 sachets of No. 4 nutritional preparation, 45-60 g every 1 to 2 h, mixed with warm water in a ratio of 1:2, taken slowly according to the principle of starting “from less to more”. At the same time, parenteral nutrition support was suspended. On postoperative day 8, patients who recovered smoothly without anastomotic leakage started eating a semi-liquid diet, such as rice noodles, custard, and gruel, and gradually transitioned to a normal diet.

After discharge from the hospital, the intervention team again carried out nutritional education with the patients and instructed them on how to have a balanced diet. In general, frequent small meals, a high-protein, high-vitamin, high-energy diet, and regular outpatient nutrition follow-up.

Patients in the control group received routine diet management with no dietary intervention before surgery. Drinking and eating were not allowed after 22:00 the night before the day of surgery. The gastric tube and duodenal nutrition tube were placed during the operation. On postoperative day 1, total parenteral nutrition was given intravenously. If the patients had anal exhaust 2-4 days after operation, enteral nutrition support could be provided through the duodenal nutrition tube. On postoperative day 5, the patients could try drinking water, on postoperative day 14, the patients could eat a semi-liquid diet, and on postoperative day 21, the patients could take regular food.

### Assessment

The primary outcome parameters included nutritional status and 30-day postoperative complications. Nutritional status included total protein and albumin levels (measured on the 2nd day of admission and the 1st, 3rd, and 7th days after operation), weight, and BMI (measured on admission day, 7th day after operation, and discharge day). Postoperative complications included pulmonary infection [according to the clinical diagnostic criteria for pulmonary infection established by the Respiratory Medicine Branch of the Chinese Medical Association (13), pulmonary infection can be diagnosed by any 4 of the following 5 items within 3 days after surgery: a) peripheral blood white blood cell count higher than 15×109/L; b) temperature ≥38°C; c) cough, expectoration, or symptoms of the original respiratory disease aggravated after the operation; d) rales heard on lung auscultation; and e) chest X-ray image showing characteristics of lung infiltrates), postoperative anastomotic fistula (diagnosed by clinical manifestations, laboratory examinations, enhanced CT, and other imaging combined diagnosis (14)] and hypoproteinemia (serum albumin concentration less than 30 g/L).

The secondary outcome parameters were intraoperative conditions (operation time, intraoperative blood loss) and gastrointestinal function recovery indicators (postoperative anal exhaust time and gastrointestinal adverse reactions such as nausea, vomiting, abdominal distension, diarrhea). Data on hospitalization costs and length of stay were also collected.

### Statistical analysis

Data were analyzed using the SPSS version 23.0 (IBM, USA). Baseline data are reported as means±SD. Independent samples *t*-test was used for comparison between two groups. Categorical variables were compared using the χ^2^ tests. P<0.05 was considered statistically significant.

## Results

### Patient characteristics

Table 1 summarizes the patient characteristics of the two groups. There was no significant difference between the two groups in sex, age, histologic subtype, location of tumor, stage, preoperative treatment, preoperative weight and BMI, preoperative white blood cell count, preoperative hemoglobin and serum albumin, thoracic approach, and abdominal approach.

**Table 1:** Clinicopathological characteristics of patients of the experimental and control groups.

Variables	Experimental group (n=120)	Control group (n=119)	P value
Age	63.78±9.13	64.12±7.91	0.759
Gender, male/female (n, %)	97 (81)/23 (19)	94 (79)/25 (21)	0.722
Histologic subtype (n, %)			0.508
Squamous cell carcinoma	106 (88)	110 (92)	
Adenocarcinoma	8 (7)	6 (5)	
Others	6 (5)	3 (3)	
Location of tumor (n, %)			0.921
Cervical esophagus	0 (0)	0 (0)	
Upper thoracic esophagus	7 (6)	5 (4)	
Mid-thoracic esophagus	72 (60)	74 (62)	
Lower thoracic esophagus	35 (29)	33 (28)	
Esophagogastric junction	6 (5)	7 (6)	
Stage (n, %)			0.881
0 and I	38 (32)	39 (33)	
II	16 (13)	12 (10)	
III	60 (50)	61 (51)	
IV	6 (5)	7 (6)	
Preoperative treatment (n, %)			0.866
None	18 (15)	22 (18)	
Chemotherapy	10 (8)	8 (7)	
Radiotherapy	6 (5)	5 (4)	
Chemoradiotherapy	86 (72)	84 (71)	
Preoperative body weight (kg)	59.44±9.10	59.57±9.13	0.912
Preoperative BMI	22.40±2.69	22.80±3.98	0.363
Preoperative WBC count (cells/L)	5.72±0.16	5.91±1.9	0.276
Preoperative hemoglobin (g/L)	132±14	134±13	0.254
Preoperative serum albumin (g/L)	42.49±5.06	42.20±5.78	0.583
Thoracic approach (n, %)			0.432
Thoracotomy	14 (12)	18 (15)	
Thoracoscopy	106 (88)	101 (85)	
Abdominal approach (n, %)			0.768
Laparotomy	6 (5)	5 (4)	
Laparoscopy	114 (95)	114 (96)	

Data are reported as means±SD or number and percent. Independent samples *t*-test or χ^2^ test. BMI: body mass index, WBC: white blood cell.

### Nutritional status

The preoperative and postoperative nutritional status was compared between the two groups. Experimental group patients had higher total protein levels than the control group patients on the 3rd (P=0.003) and 7th (P<0.001) days after surgery. Experimental group patients had higher albumin levels than control group patients on the 3rd (P=0.028) and 7th days (P=0.005) after surgery. There were no statistically significant differences in weight and BMI between the experimental group and the control group on the day of admission, the 7th day after surgery, and the day of discharge (P>0.05) (Table 2).

**Table 2:** Comparison of preoperative and postoperative nutritional status between the experimental and control groups.

Variables	Experimental group (n=120)	Control group (n=119)	*t* value	P value
Total protein (g/L)				
Admission	68.28±5.06	67.84±5.78	0.62	0.534
Day 1 after surgery	56.80±4.39	57.47±5.14	-1.07	0.284
Day 3 after surgery	60.53±5.23	58.44±5.57	3.00	0.003
Day 7 after surgery	64.77±5.72	61.65±5.91	4.150	<0.001
Albumin (g/L)				
Admission	42.49±5.06	42.20±5.78	0.55	0.583
Day 1 after surgery	33.60±3.81	34.10±5.19	-0.85	0.397
Day 3 after surgery	36.40±3.86	35.07±5.37	2.21	0.028
Day 7 after surgery	38.52±4.36	36.52±6.29	2.86	0.005
Weight (kg)				
Admission	59.44±9.10	59.57±9.13	-0.11	0.913
Day 7 after surgery	59.20±9.10	59.05±8.88	0.13	0.901
Discharge	58.28±10.17	58.16±8.77	0.10	0.920
BMI				
Admission	22.40±2.69	22.80±3.98	-0.92	0.361
Day 7 after surgery	22.32±2.73	22.35±3.23	-0.09	0.932
Discharge	22.11±2.72	22.00±3.24	0.283	0.778

Data are reported as means±SD. Student's *t*-test. BMI: body mass index.

### Postoperative complications

Table 3 summarizes the postoperative complications of the two groups. Pulmonary infection was diagnosed in 9 patients (7.50%) in the experimental group and 34 patients (28.57%) in the control group (P<0.001). Postoperative anastomotic fistula was diagnosed in 1 patient (0.83%) in the experimental group and 11 patients (9.24%) in the control group (P=0.003). On the 3rd day after surgery, hypoproteinemia was diagnosed in 58 patients (48.33%) in the experimental group and 73 patients (61.34%) in the control group (P<0.001). On the 7th day after surgery, hypoproteinemia was diagnosed in 20 patients (16.67%) in the experimental group and 52 patients (56.57%) in the control group (P<0.001) (Table 3).

**Table 3:** Comparison of postoperative complications between experimental and control groups.

Variables	Experimental group (n=120)			Control group (n=119)		χ^2^ value	P value
	Yes	No		Yes	No		
Pulmonary infection	9 (7.50%)	111 (92.50%)		34 (28.57%)	85 (71.43%)	17.980	**<0.001**
Anastomotic fistula	1 (0.83%)	119 (99.17%)		11 (9.24%)	108 (90.76%)	8.86	**0.003**
Hypoproteinemia							
Day 1 after surgery	91 (75.83%)	29 (24.17%)		86 (72.27%)	33 (27.73%)	0.395	0.530
Day 2 after surgery	58 (48.33%)	62 (51.67%)		73 (61.34%)	46 (38.66%)	20.568	**<0.001**
Day 3 after surgery	20 (16.67%)	100 (83.33%)		52 (43.33%)	67 (56.57%)	24.579	**<0.001**

Data are reported as numbers (percentages). χ^2^: chi-squared test. Significant P values are indicated in bold type.

### Intraoperative conditions

The operation time of patients in the experimental group (3.96±0.80 h) was lower than that in the control group (4.55±1.07 h) (P<0.001). Intraoperative blood loss in the experimental group (44.33±31.91 mL) was less than that in the control group (77.31±53.91 mL) (P<0.001).

### Postoperative gastrointestinal function recovery

The postoperative anal exhaust time in the experimental group (2.93±1.13 d) was lower than that in the control group (4.81±1.52 d) (P<0.001). The incidence of postoperative gastrointestinal adverse reactions in the experimental group (5%) was lower than that in the control group (32.77%) (P<0.001). Among them, the incidence of nausea (1.67%), vomiting (1.67%), and abdominal distension (0.83%) in the experimental group was lower than the incidence of nausea (10.92%), vomiting (9.24%), and abdominal distension (21.01%) in the control group (P=0.003, P=0.010, P<0.001, respectively).

### Hospitalization costs

The total cost of hospitalization for the experimental group (US$ 10,880.20±2,933.83) was lower than that of the control group (US$ 12,044.64±4,446.74) (P=0.018). There was no statistically significant difference in the length of hospitalization between the two groups (P=0.806).

## Discussion

The MDT model is a clinical diagnosis and treatment model in which experts from more than 2 related disciplines form a fixed expert group who provide diagnosis and treatment opinions for a certain disease through regular meetings (15). This model had become an important model for diagnosis and treatment of diseases in many hospitals worldwide. Previous studies have confirmed that the MDT model can improve the prognosis, quality of life, and survival of patients (4,9,11). The MDT model played an important role in enhanced recovery after surgery (ERAS). In recent years, the MDT model has rapidly developed in Chinese hospitals. Because the risk of malnutrition in patients with esophageal cancer is high and the patients' nutritional status is an independent factor predicting patient survival after surgery, the MDT model was applied to the nutritional management of patients in this study.

Traditional perioperative nutritional management has some limitations. First, it lacks preoperative nutritional status assessment and intervention, missing the opportunity to improve the nutritional status of patients before surgery. Previous studies have confirmed that nutritional assessment and targeted intervention of patients before surgery could improve patients' tolerance to surgery, enhance patients' immunity, and reduce the incidence of postoperative complications (16-18). Second, not being allowed to drink or eat for the required time before surgery affects the patient's mood and aggravates the stress on the body during surgery. The purpose of fasting before surgery is to prevent aspiration pneumonia caused by vomiting after anesthesia. However, previous studies have confirmed that under normal circumstances, it takes 6 h to empty the stomach of solid foods and only 2 h of liquids. Surgery after fasting overnight is equivalent to climbing a mountain or running in a hungry state, which causes stress and greatly disturbs the body's homeostasis. Therefore, the ERAS concept does not advocate long-term fasting. It is recommended that patients take sugar-containing liquids before surgery. Third, patients with conventional gastric tube and nutrition tube take longer to transition to oral feeding, which is not conducive to the rapid recovery of patients after surgery. The gastrointestinal decompression tube and duodenal nutrition tube placed after the operation remains as a foreign body in the patient's body (19), which increases the infection rate of the patient and leads to psychological disorders, sleep disorder, unplanned extubation, etc. (20). Moreover, the tube limits the patients' postoperative activities, resulting in an increased incidence of postoperative abdominal distension, pulmonary infection, and venous thrombosis (21). Previous studies have also confirmed that gastrointestinal decompression neither effectively extracts digestive juices to reduce tension in the gastrointestinal tract nor prevents aspiration caused by reflux of gastric contents (22,23). On the contrary, the nasogastric tube would affect the function of the lower esophageal sphincter, associated with varying degrees of damage to the swallowing muscles during the operation, and affect the postoperative physiological anatomy. Patients with nasogastric tubes are more likely to have gastric reflux, which may cause lung infections due to improper aspiration. One study has demonstrated that oral energy supply in the early postoperative period can accelerate recovery of gastrointestinal function, improve the nutritional status and quality of life of patients without increasing postoperative complications, and thereby shorten the length of hospital stay (24).

The strength of MDT perioperative nutrition management is the multidisciplinary expert collaboration, the preoperative nutritional assessment of patients, and the development of professional and individualized nutritional programs. Patients in the MDT group were allowed to eat 2-4 h before surgery. The gastric tube and nutrition tube were not installed during the operation. Patients were able eat by mouth early after surgery. This series of measures improved the nutritional status of patients during the perioperative period. Evidence shows that the nutritional status of the human body is closely related to the content of coagulation factors such as vitamin K in the body (25,26). The improvement of the patient's preoperative nutritional status can improve the coagulation function, which can reduce bleeding during the operation and thus reduce operation time. In addition, a previous study found that preoperative nutritional support can enhance the patient's surgical tolerance and improve the postoperative nutritional status (27).

There were no differences in serum total protein and albumin levels and in the incidence of hypoalbuminemia between the groups on the 1st postoperative day. The reason for this may be that the huge impact of surgery on protein metabolism masked the effect of the experimental intervention on the patients. However, the protein recovery on the 3rd and 7th days after surgery was better, and the incidence of hypoalbuminemia was also lower in the experimental group than in the control group.

In conclusion, MDT nutrition management could effectively improve the nutriture of patients, promote the rapid recovery of postoperative gastrointestinal function, reduce postoperative complications, and reduce hospitalization costs.

## References

[1] 1. Bray F, Ferlay J, Soerjomataram I, Siegel RL, Torre LA, Jemal A. Global cancer statistics 2018: GLOBOCAN estimates of incidence and mortality worldwide for 36 cancers in 185 countries. CA Cancer J Clin 2018; 68: 394-424, doi: 10.3322/caac.21492. 10.3322/caac.21492 30207593

[2] 2. Lin Y, Totsuka Y, Shan B, Wang C, Wei W, Qiao Y, et al. Esophageal cancer in high-risk areas of China: research progress and challenges. Ann Epidemiol 2017; 27: 215-221, doi: 10.1016/j.annepidem.2016.11.004. 10.1016/j.annepidem.2016.11.004 28007352

[3] 3. Filip B, Scarpa M, Cavallin F, Cagol M, Alfieri R, Saadeh L, et al. Postoperative outcome after oesophagectomy for cancer: Nutritional status is the missing ring in the current prognostic scores. Eur J Surg Oncol 2015; 41: 787-794, doi: 10.1016/j.ejso.2015.02.014. 10.1016/j.ejso.2015.02.014 25890494

[B04] 4. Steenhagen E, van Vulpen JK, van Hillegersberg R, May AM, Siersema PD. Nutrition in peri-operative esophageal cancer management. Expert Rev Gastroenterol Hepatol 2017; 11: 663-672, doi: 10.1080/17474124.2017.1325320. 10.1080/17474124.2017.1325320 28454509

[5] 5. Steenhagen E. Preoperative nutritional optimization of esophageal cancer patients. J Thorac Dis 2019; 11: S645-S653, doi: 10.21037/jtd.2018.11.33. 10.21037/jtd.2018.11.33 PMC6503268 31080641

[6] 6. Bozzetti F, Mariani L, Lo Vullo S, Amerio ML, Biffi R, Caccialanza G, et al. The nutritional risk in oncology: a study of 1,453 cancer outpatients. Support Care Cancer 2012; 20: 1919-1928, doi: 10.1007/s00520-012-1387-x. 10.1007/s00520-012-1387-x PMC3390688 22314972

[7] 7. Heneghan HM, Zaborowski A, Fanning M, McHugh A, Doyle S, Moore J, et al. Prospective study of malabsorption and malnutrition after esophageal and gastric cancer surgery. Ann Surg 2015; 262: 803-807; discussion 807-808, doi: 10.1097/SLA.0000000000001445. 10.1097/SLA.0000000000001445 26583669

[8] 8. Toyokawa T, Kubo N, Tamura T, Sakurai K, Amano R, Tanaka H, et al. The pretreatment Controlling Nutritional Status (CONUT) score is an independent prognostic factor in patients with resectable thoracic esophageal squamous cell carcinoma: results from a retrospective study. Bmc Cancer 2016; 16: 722, doi: 10.1186/s12885-016-2696-0. 10.1186/s12885-016-2696-0 PMC5013653 27599460

[9] 9. Marchi F, Filauro M, Missale F, Parrinello G, Incandela F, Bacigalupo A, et al. A multidisciplinary team guided approach to the management of cT3 laryngeal cancer: a retrospective analysis of 104 cases. Cancers (Basel) 2019; 11: 717, doi: 10.3390/cancers11050717. 10.3390/cancers11050717 PMC6562846 31137671

[10] 10. Shirakawa Y, Noma K, Maeda N, Tanabe S, Sakurama K, Sonoyama-Hanaoka A, et al. Early intervention of the perioperative multidisciplinary team approach decreases the adverse events during neoadjuvant chemotherapy for esophageal cancer patients. Esophagus 2021; 18: 797-805, doi: 10.1007/s10388-021-00844-y. 10.1007/s10388-021-00844-y 33999305

[11] 11. Sun K, Goodfellow H, Konstantara E, Hill A, Lennard D, Lloyd-Dehler E, et al. The multidisciplinary, theory-based co-design of a new digital health intervention supporting the care of oesophageal cancer patients. Digit Health 2021; 7: 20552076211038410, doi: 10.1177/20552076211038410. 10.1177/20552076211038410 PMC8642779 34873450

[12] 12. Bauer J, Capra S, Ferguson M. Use of the scored Patient-Generated Subjective Global Assessment (PG-SGA) as a nutrition assessment tool in patients with cancer. Eur J Clin Nutrit 2002; 56: 779-785, doi: 10.1038/sj.ejcn.1601412. 10.1038/sj.ejcn.1601412 12122555

[13] 13. Li S, Su J, Sui Q, Wang G. A nomogram for predicting postoperative pulmonary infection in esophageal cancer patients. Bmc Pulm Med 2021; 21: 283, doi: 10.1186/s12890-021-01656-7. 10.1186/s12890-021-01656-7 PMC8422704 34488717

[14] 14. Girard E, Messager M, Sauvanet A, Benoist S, Piessen G, Mabrut JY, et al. Anastomotic leakage after gastrointestinal surgery: diagnosis and management. J Visc Surg 2014; 151: 441-450, doi: 10.1016/j.jviscsurg.2014.10.004. 10.1016/j.jviscsurg.2014.10.004 25455960

[15] 15. Sanal G, Shijin S, Krishna V, Kesavadev J, Basanth A, Krishnan G, et al. Empowering patients with type 1 diabetes through a multidisciplinary team-assisted, technology-enabled education program. Curr Diabetes Revi 2022. doi: 10.2174/1573399818666220520115420. 10.2174/1573399818666220520115420 35619301

[16] 16. Kobayashi K, Koyama Y, Kosugi S, Ishikawa T, Sakamoto K, Ichikawa H, et al. Is early enteral nutrition better for postoperative course in esophageal cancer patients? Nutrients 2013; 5: 3461-3469, doi: 10.3390/nu5093461. 10.3390/nu5093461 PMC3798914 24067386

[B17] 17. Cao S, Zhao G, Cui J, Dong Q, Qi S, Xin Y, et al. Fast-track rehabilitation program and conventional care after esophagectomy: a retrospective controlled cohort study. Support Care Cancer 2013; 21: 707-714, doi: 10.1007/s00520-012-1570-0. 10.1007/s00520-012-1570-0 22933129

[18] 18. Fujita T, Daiko H, Nishimura M. Early enteral nutrition reduces the rate of life-threatening complications after thoracic esophagectomy in patients with esophageal cancer. Eur Surg Res 2012; 48: 79-84, doi: 10.1159/000336574. 10.1159/000336574 22377820

[19] 19. Sun H, Li Y, Liu X, Wang Z, Zhang R, Qin J, et al. Feasibility of “no tube no fasting” therapy in thoracolaparoscopic oesophagectomy for patients with oesophageal cancer [in Chinese]. Zhonghua Wei Chang Wai Ke Za Zhi 2014; 17: 898-901. 25273659

[20] 20. Blumenstein I, Shastri YM, Stein J. Gastroenteric tube feeding: techniques, problems and solutions. World J Gastroenterol 2014; 20: 8505-8524, doi: 10.3748/wjg.v20.i26.8505. 10.3748/wjg.v20.i26.8505 PMC4093701 25024606

[21] 21. Bauer VP. The evidence against prophylactic nasogastric intubation and oral restriction. Clin Colon Rectal Surg 2013; 26: 182-185, doi: 10.1055/s-0033-1351136. 10.1055/s-0033-1351136 PMC3747290 24436672

[22] 22. Prabhakaran S, Doraiswamy VA, Nagaraja V, Cipolla J, Ofurum U, Evans DC, et al. Nasoenteric tube complications. Scand J Surg 2012; 101: 147-155, doi: 10.1177/145749691210100302. 10.1177/145749691210100302 22968236

[23] 23. Sun HB, Li Y, Liu XB, Wang ZF, Zhang RX, Lerut T, et al. Impact of an early oral feeding protocol on inflammatory cytokine changes after esophagectomy. Ann Thoracic Surg 2019; 107: 912-920, doi: 10.1016/j.athoracsur.2018.09.048. 10.1016/j.athoracsur.2018.09.048 30403976

[24] 24. Willcutts KF, Chung MC, Erenberg CL, Finn KL, Schirmer BD, Byham-Gray LD. Early oral feeding as compared with traditional timing of oral feeding after upper gastrointestinal surgery: a systematic review and meta-analysis. Ann Surg 2016; 264: 54-63, doi: 10.1097/SLA.0000000000001644. 10.1097/SLA.0000000000001644 26779983

[25] 25. Weimann A, Braga M, Carli F, Higashiguchi T, Hübner M, Klek S, et al. ESPEN guideline: Clinical nutrition in surgery. Clin Nutr 2017; 36: 623-650, doi: 10.1016/j.clnu.2017.02.013. 10.1016/j.clnu.2017.02.013 28385477

[26] 26. Card DJ, Gorska R, Harrington DJ. Laboratory assessment of vitamin K status. J Clin Pathol 2020; 73: 70-75, doi: 10.1136/jclinpath-2019-205997. 10.1136/jclinpath-2019-205997 31862867

[27] 27. Haneda R, Hiramatsu Y, Kawata S, Honke J, Soneda W, Matsumoto T, et al. Survival impact of perioperative changes in prognostic nutritional index levels after esophagectomy. Esophagus 2022; 19: 250-259, doi: 10.1007/s10388-021-00883-5. 10.1007/s10388-021-00883-5 PMC8921021 34546503

